# Near-instant automatic access to visually presented words in the human neocortex: neuromagnetic evidence

**DOI:** 10.1038/srep26558

**Published:** 2016-05-24

**Authors:** Yury Shtyrov, Lucy J. MacGregor

**Affiliations:** 1 Center of Functionally Integrative Neuroscience (CFIN), Department of Clinical Medicine, Aarhus University, Aarhus, Denmark; 2 Centre for Cognition and Decision Making, National Research University Higher School of Economics, Moscow, Russia; 3 Medical Research Council Cognition and Brain Sciences Unit, Cambridge, UK

## Abstract

Rapid and efficient processing of external information by the brain is vital to survival in a highly dynamic environment. The key channel humans use to exchange information is language, but the neural underpinnings of its processing are still not fully understood. We investigated the spatio-temporal dynamics of neural access to word representations in the brain by scrutinising the brain’s activity elicited in response to psycholinguistically, visually and phonologically matched groups of familiar words and meaningless pseudowords. Stimuli were briefly presented on the visual-field periphery to experimental participants whose attention was occupied with a non-linguistic visual feature-detection task. The neural activation elicited by these unattended orthographic stimuli was recorded using multi-channel whole-head magnetoencephalography, and the timecourse of lexically-specific neuromagnetic responses was assessed in sensor space as well as at the level of cortical sources, estimated using individual MR-based distributed source reconstruction. Our results demonstrate a neocortical signature of automatic near-instant access to word representations in the brain: activity in the perisylvian language network characterised by specific activation enhancement for familiar words, starting as early as ~70 ms after the onset of unattended word stimuli and underpinned by temporal and inferior-frontal cortices.

The human brain has evolved to support rapid information processing, enabling us to react quickly and efficiently to events in the constantly changing world around us, a skill vital to our biological survival. Our key communication tool, language, constitutes a highly dynamic stream of information which needs to be processed by the central nervous system with minimal delay, in order to ensure its timely comprehension and to guarantee optimal reactions to any potentially important messages. Indeed, the known neuroanatomical and functional connectivity^1^,^2^,^3^,^4^,^5^,^6^ can support very high speeds of information transfer, in principle allowing for signal conduction from peripheral sensory input to the language cortices in temporal and frontal lobes at delays well below 1/10 of a second. However, the evidence of linguistically specific neural processes at these early latencies remains scarce. A key component of linguistic processing is lexical access, that is, the mapping of perceived words onto long-term memory representations, resulting in the access of the word form (a sort of “entry” in the brain’s “mental lexicon”) and the semantic information (“meaning”) it carries. Recent research has suggested that, when listening to spoken words, a perisylvian network of key brain regions dealing with language may be automatically ignited within ~30–80 milliseconds of the information arriving at the ear, thus providing a neurobiological substrate for rapid linguistic processing^7^,^8^,^9^. These high speed and automaticity were attributed to the robustness of distributed neuronal networks that act as neural word memory traces in the brain^10^. However, such suggestions of ultra-rapid word processing cannot be adequately verified in the auditory domain: in spoken language, words unfold in time, and therefore word-initial information allows for predictive processing and pre-activation of corresponding memory traces prior to word completion and even before the word recognition point^11^. This could lead to earlier brain responses thus potentially confounding any conclusions on the temporal dynamics of neural word comprehension^12^. In contrast to this, in the visual domain, sensory information about the word is available instantaneously and in its entirety (for short words at least). Using visually-presented words could therefore help parcel out the earliest stages of neural word access unconfounded by predictive effects, by avoiding the complications associated with time-locking brain responses to information that unfolds over time. Equally importantly, as language is essentially a multi-modal neurocognitive function (involving at least auditory and visual inputs), the rapid automatic activation of word memory traces could be predicted to take place upon *any* presentation of linguistic information, irrespective of the modality in which it is presented. However, until now, such rapid neurolexical activity for visually presented language has not been observed. Previous studies have mostly focussed on brain responses in a much later latency range [typically hundreds of milliseconds; see e.g.^13^]; the earliest known lexically-specific neurophysiological effects have been found to build up by ~160 ms^14^, which is still considerably later than both predicted by conduction time estimates and found in the auditory domain. Here, we therefore addressed the earliest stages of visual word processing, understanding of which is essential for comprehensive understanding of multimodal neurobiological mechanisms underlying the language function.

We investigated the neural time course of lexical access to written language by comparing neuromagnetic brain responses to a large set of distinct meaningful words with a matched set of word-like orthographically regular and phonotactically legal meaningless word forms (“pseudowords”) that were rigorously balanced on psycholinguistic and physical properties and thus differed only in terms of their lexical status. To probe the putative automaticity in neural access of visually presented language, the stimuli were presented tachistiscopically on the periphery of the visual field whilst participants’ attention was diverted from the parafoveal linguistic stimuli to a non-linguistic dual feature-detection task. Such a non-attend design also helps to rule out potential masking effects that focused attention is known to have on the earliest neurophysiological correlates of word access^15^,^16^. Neuromagnetic brain activity was recorded using a high-density whole-head MEG set-up, and event-related fields and their underlying cortical generators (based on single-subject neuroanatomical brain surfaces) were estimated. Based on the above theoretical assumptions and neuroanatomical conduction time estimates, we predicted a rapid activation of language-specific areas in the brain even by such unattended linguistic stimuli, which would automatically dissociate between the meaningful and senseless language inputs. Previous investigations of structural^6^,^17^,^18^ and functional^4^,^19^,^20^ connectivity in the language function suggested an orderly temporo-frontal activation dynamics (with delays of a few tens of milliseconds between key linguistic areas), particularly expressed in the language-dominant left hemisphere. A similar sequence could therefore also be expected at the putative ultra-early neural stage of visual word access; we therefore focussed on scrutinising the dynamics of early brain activation arising from the vicinity of perisylvian core language areas.

## Results

**Figure 1:**
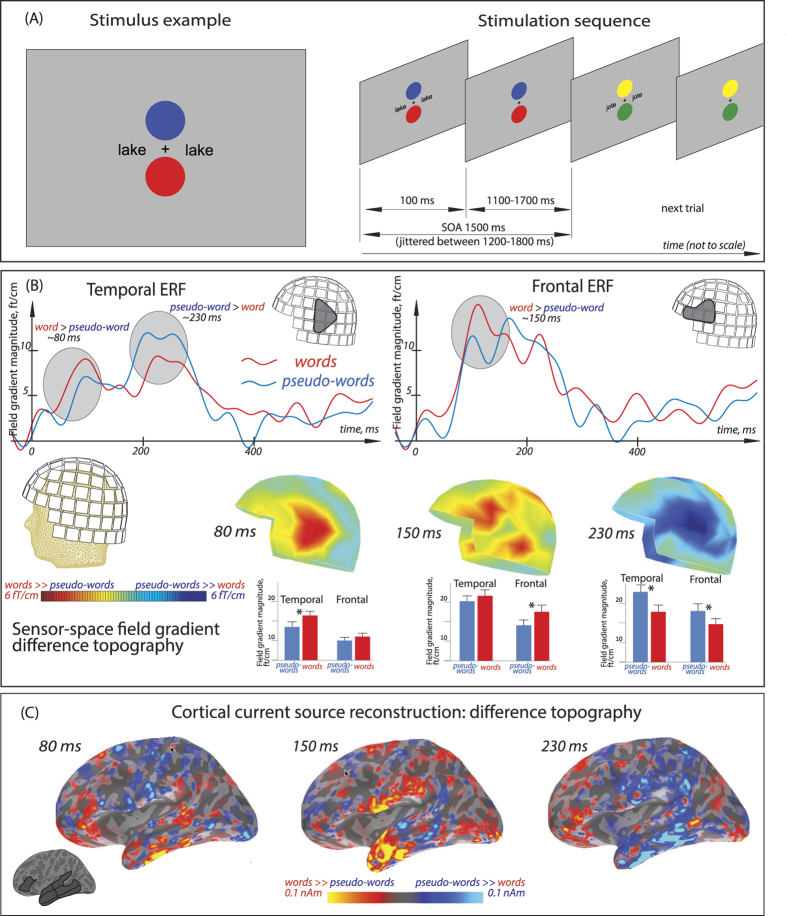
(**A***, top*). An example of visual stimulus and stimulation sequence. The participants were instructed to track colour/location combinations of circles that were continuously present on the screen, while matched words or pseudowords were tachistoscopically flashed at unattended symmetric locations at the same time when the non-linguistic stimuli changed colour, ensuring maximal distraction from linguistic information. (**B***, middle*). Grand average MEG responses (magnetic gradient RMS amplitudes) over the left posterior and anterior areas. Both waveforms and difference topographies of event-related responses indicated differential brain activation in response to unattended meaningful words and meaningless pseudowords, starting with the first difference in the temporal sensors already at 70–90 ms and continuing until ~250 ms post-onset. Schematic MEG helmet diagrams illustrate the selection of sensor clusters used to separate the relatively more temporal and frontal ERF dynamics. (**C***, bottom*). Grand average word-pseudoword contrast in MEG source space (minimum-norm estimates on inflated cortical surface based on single-subject MR images) confirmed early processing of lexical contrasts in temporal and inferior-frontal neocortex. A schematic diagram based on average cortical surface illustrates the atlas-based neuroanatomical ROIs used for extracting source amplitudes.

All subjects successfully performed the behavioural dual visual feature-detection task (identification of colour/location combinations). Average hit rate was 96.4% (range: 83.3–100.0%), with false alarm rate of only 1.88% (range: 0.0–4.6%) and average d′ of 0.95, indicating that the experimental design directing the subjects’ attention onto this non-linguistic task was successful. At the same time, MEG recordings produced expressed event-related neuromagnetic responses, which were, however, notably different between linguistic stimulus types. Even though the word and pseudoword stimuli were not attended, and were matched for a range of physical and psycholinguistic factors, the brain’s activation to them showed a tri-phasic difference in the sensor channels located above left-hemispheric perisylvian areas (Fig. 1B). Already in the 70–90 ms analysis window, left temporal gradiometers indicated a significant enhancement of responses to unattended words over those to pseudowords (F(1, 17) = 16.9, p = 0.0007). At 140–160 ms the significant lexical enhancement effect progressed to the more anterior region-of-interest (ROI; F(1, 18) = 7.08, p = 0.016). Finally, at a later 240–260 ms time window, the opposite effect - wide-spread increase for pseudoword-elicited activation was found (F(1, 17) = 9.77, p = 0.006) - and was significant at both temporal (F(1, 17) = 6.99, p = 0.017) and frontal (F(1, 17) = 7.19, p = 0.016) sensor ROIs. While a trend for similar effects appeared to be also present in the right hemisphere, no significant results were obtained for the right homologue ROIs; this divergence between left- and right-hemispheric patterns was confirmed by a significant Lexicality X Hemisphere interaction (F(1, 17) = 4.64, p = 0.046). Crucially, no differences could be seen in occipital sensors (tested ad hoc), implying that the intended matching of stimuli’s visual physical properties was successful and thus the basic visual sensory activation per se could not bias the response pattern.

These sensor level analyses were followed up with cortical source statistics, which indicated a similar pattern of results. At the earliest window, the Lexicality effect (increased word response) was significant in the temporal lobe ROI as a whole (F(1, 17) = 4.64, p = 0.045; no interactions or main effects arose involving subdivisions of temporal ROI) confirming the sensor-space result. Similar to the frontal ROI in sensor space, source analysis of the inferior-frontal gyrus as a whole did not yet show lexicality effect at this earliest time frame. These differences between temporal and frontal area activations were also reflected in a significant interaction Area X Lexicality (F(1, 17) = 5.364, p = 0.033). Interestingly, however, IFG showed an interaction between sub-ROI and Lexicality factors (F(1, 17) = 5.076, p = 0.038); planned comparisons indicated that this was due to significant amplitude advantage (word > pseudoword) in pars orbitalis only (F(1, 17) = 6.174, p = 0.024) but not elsewhere in the IFG. At the second window, no main effects reached significance in source space, but an interaction arose between temporal sub-ROI and Lexicality factors (F(1, 17) = 3.891, p = 0.05); planned comparisons indicated that this was due to significantly larger amplitude for word than pseudoword responses in the anterior-temporal cortex only (F(1, 17) = 6.409, p = 0.022), but not in the posterior-temporal areas. Finally, in the last time window (220–240 ms), the larger pseudoword than word response that was found to be fully significant in signal space, was confirmed as a marginally significant difference in the left temporal activation (F(1, 17) = 3.78, p = 0.069).

## Discussion

To elucidate the putative early timecourse of automatic visual word processing in the brain, we recorded automatic neuromagnetic brain responses to psycholinguistically and visually matched words and pseudowords that were briefly presented parafoveally to participants outside their focus of attention while they were concentrating on a non-linguistic dual visual task. The results indicated a rapid and automatic tri-phasic timecourse, with word-specific response enhancement in temporo-frontal neocortical regions between ~70 and ~160 ms after stimulus onset, and a later response increase to meaningless pseudoword items at 220–240 ms. We will briefly consider these findings below.

The first striking result is the *earliness* with which lexical differences are reflected in the brain activation. The word-pseudoword difference became exhibited as an *increased word activation* that started from 70 ms after presentation. As words and pseudowords were matched for physical, orthographic and psycholinguistic features, it is unlikely that it was driven by low-level perceptual differences. Instead, we suggest that the effect is due to lexical familiarity - the presence of established memory representations for the familiar meaningful word stimuli. The lexical ERP/ERF enhancement, which has been observed previously in the auditory modality, has been explained in terms of the activation of memory representations in the brain for real words as opposed to the purely sensory activation which occurs for meaningless pseudowords that do not possess memory representations^21^,^22^. Here, the word advantage became obvious in sensor space already at 70–90 ms; the source analysis suggested its origins to be in the temporal lobe as well as in pars opercularis, an integral part of what traditionally is known as Broca’s area. This speed is much faster than any previously reported lexically-specific brain reactions to visual words, the earliest of which have been known to build up by ~160 ms^14^. At the same time, this latency corresponds well to our theoretical estimates of axonal conduction delay between peripheral sensors and cortical associative areas (below 100 ms), and is in fact somewhat delayed if compared with the earliest estimates of spoken word recognition known to occur already at 30–60 ms^7^,^8^,^23^. This relative delay in perisylvian activation for visual words is likely explained by an additional step of transferring information from occipital visual to language-specific temporal cortices (that are located in immediate vicinity of auditory cortex, reducing the transfer time for spoken language). An important difference between the previous spoken word investigations and the current study is that here the word-specific response is time-locked to the visual stimulus onset, whereas in the auditory modality, where stimuli unfold over time, the early lexicality effects were time locked to post-onset word recognition points. Thus, whereas in the auditory domain, rapid brain reflections of words’ lexico-semantic properties could be at least in part explained by partial pre-activation of memory traces based on incomplete acoustic information prior to the recognition points (in line with the Cohort model of speech recognition^11^), this confound is fully excluded for the present visual data, where the brain responses start to diverge at 70 ms after the word onset. These data therefore suggest that our brain may be capable of near-instantaneous access to information about the words it encounters, a capability that is important to the efficient and reliable use of language as our primary communication tool.

The second striking result is the *automaticity* of these word and pseudoword brain responses. While the presentation itself did not exclude words from entering visual input, the visual dual feature detection task made it rather difficult for the subjects to pay active attention to very brief (100 ms only) presentation of words in parafoveal areas. This very much mimicked an everyday situation where we may catch a glimpse of roadside commercial billboards whilst focusing on driving, or be influenced by newspaper or online adverts outside our visual focus without having any intention of reading them. Not only does such briefly and parafoveally presented language appear to enter the visual sensory system, but, as our results suggest, it is processed in language-specific temporo-frontal cortical networks, likely leading to an activation of neural memory circuits for individual words.

Previous studies using masked priming paradigm have also found lexico-semantic effects dependent on ‘invisible’ prime words [e.g.^24^,^25^,^26^], albeit their EEG correlates have largely been located in a much later time frame, predominantly in the 400 ms range [e.g.^27^,^28^]. An important difference, however, is that in the masked priming designs the stimuli are usually attended in an active linguistic task (e.g. lexical decision), even though they may escape awareness through masking manipulation. The same is true for behavioural studies of automatic language processing using Stroop effects [e.g.^26^,^29^,^30^], where, even if the task does not encourage reading per se, multi-colour word stimuli are presented in the focus of attention. Here, instead, the stimuli are outside the focus of attention while the subjects’ task is strictly non-linguistic and does not encourage attentive linguistic processing in any way. Further, while the priming paradigm is typically aimed at revealing relationships between the prime and the probe stimuli, here we address the processing of unattended stimulus per se and show that lexical familiarity significantly affects brain responses to such stimuli. Taken together, the current result appears to provide evidence of automatic processing of unattended written language with lexical memory trace activation/access taking place even when this is irrelevant for task requirements and when attention is diverted away from written words.

The speed and automaticity of processing therefore seem to be properties that are shared by both spoken and written language access, and thus appear to constitute a supramodal mechanism used in auditory and visual modalities alike. Whereas reading is a very recent addition to the human communicative inventory, visual object recognition, on the other hand, is an evolutionary ancient and well perfected skill underpinned by highly developed cortical systems^31^ which might well be employed in letter and word recognition^32^,^33^. Thus, our ability to rapidly and automatically assess potentially important visually presented messages should not really be surprising. Future studies are, however, needed to address in more detail the depth of such automatic processing and, for instance, investigate the entire range of exact features (e.g., semantic, morphosyntactic, phonological) that may become activated in such non-attend visual presentation mode.

Finally, the brain response pattern has shown clear *spatio-temporal dynamics* of these automatic neurolexical processes. Signal-space analysis suggested the first phase of lexically-specific activity could be generated in the left temporal lobe, which was confirmed by similar (albeit not identical) source-space results. To establish that this is not a carry-over of any purely visual differences between stimulus types, we also did an *ad hoc* comparison of activations in occipital and fusiform areas, which produced no significant contrasts. Since both words and pseudowords were well-formed orthographically and phonologically, the apparent absence of differential activity in the left fusiform cortex cannot as such speak against its specialisation for visual word forms (the so-called visual word form area, VWFA^34^), postulated by some models and refuted by others^35^. At the same time, the very fact that the earliest lexical differences to visually presented stimuli are present in temporal areas speaks in favour of the linguistic character of this rapid automatic activation. Thus, it seems to reflect a first-pass automatic activation of lexical memory representations that may have their central ‘hub’ in the temporal lobe^36^. By ~150 ms, the peak of this lexical contrast has moved more frontally in sensor space, with source analysis locating its generators in anterior-temporal areas. While the spatial resolution of MEG is limited and neuroanatomical precision of the current localisation should be treated with some caution, the statistical separation between the relatively more posterior/temporal and frontal dynamics is per se unambiguous. Importantly, this delay is in line with the theoretical framework of posterior-anterior information transfer in language processing, likely carried over the so called ventral and dorsal pathways^6^,^17^,^18^. Such a functional connection must have its substrate in anatomical connections such as arcuate and uncinate fasciculae and capsula extrema, whose role in the language function is only beginning to be understood^17^. Finally, by ~230 ms after stimulus onset, the initial word advantage was replaced by a more wide-spread activation that was stronger for meaningless pseudowords. This latter pattern is reminiscent of the so-called N400 response that, in active paradigms at least, is known to be increased for meaningless materials such as pseudowords^37^,^38^. It has been suggested to reflect enhanced processes of lexical search and repair caused by the failure of initial parsing of a senseless stimulus^39^. Crucially, the current result suggests that these processes take place even when the stimuli are not attended to or actively processed, implying that they possess a certain degree of automaticity^40^. At the same time, the response does not develop into a fully-fledged N400, as no clear peak was observed at 400 ms, suggesting that these initial automatic search processes are extinguished when attention-controlled neural resources are not fully available^15^,^16^,^41^. The latter, however, remains to be elucidated in future studies that could use more typical N400 paradigms and compare them with a non-attend presentation mode employed here.

The posterior-anterior order of activation we show here is generally similar to that previously shown to develop at a later time with a somewhat lower pace in active word reading paradigms (300–400 ms^19^); it therefore possibly reflects an automatic first-pass parsing step which may be replayed at a deeper level at secondary processing stages if attention is allocated to the linguistic input. Notably, the current dynamics matches well with auditory studies using unattended single word presentation paradigms^4^,^20^, again suggesting a high degree of similarity between word processing in the two modalities. This sequence of activation is in line with current mainstream models of reading^42^,^43^ that suggest that the information from visual occipital areas may be passed in a dual-route fashion both to left-temporal lexico-semantic representations via the ventral route, and, through the dorsal pathway, to perisylvian phonological representations. That both temporal and frontal areas are activated by unattended meaningful words suggests that both lexical and phonological routes may be engaged in an automatic fashion; the exact levels of their involvement remains to be investigated in future studies.

A few further words of caution are warranted when discussing the cortical substrate and the temporal dynamics implicated by the current results. First, as we mentioned above, the spatial resolution of MEG is limited and one should be careful when interpreting neuroanatomical localisation of the present effects. This uncertainty is well illustrated by the mapping of the frontal sensor-space effect in the second time window onto anterior-temporal (and not inferior-frontal) cortex in the source space. Regardless of this, the generally similar patterns of both signal and source analysis and their correspondence with theoretical predictions and existing literature (including both auditory and visual modalities), speak in favour of interpreting the current pattern as reflecting a dynamic perisylvian activation, the precise progression of which will need to be validated in future research. Second, we have focused here on an a priori preselected set of ROIs in order to test the theoretically predicted involvement of core language areas in early automatic processing of unattended words. Although other areas have largely remained outside the scope of the current study, it certainly does not imply that they could not be involved. Indeed, as evident in the source activation landscapes, substantial activity can be seen elsewhere. Future studies could use other paradigms, as well as include more ROIs or even employ a whole-brain analysis, to comprehensively test the timecourse and the spatio-temporal dynamics of the early activity which we report here for the first time using an a priori defined small selection of areas. Finally, the analysis was optimised not only in spatial, but also in temporal terms, and geared towards early transient responses, including, e.g., filter settings, a topic of much discussion in the current literature [e.g.^44^,^45^; notably the procedures applied here are rather standard for ERF/ERP analysis and were the same for the both stimulus types, implying that no differential effects could be introduced into the two conditions that could lead to artifactual differences between them]. Future studies, using different stimuli and including different tasks (e.g., more active ones geared towards N400 elicitation) as well as analysis procedures (e.g. different filter bands and baseline intervals) optimised for electrophysiological shifts of different frequencies and temporal dynamics^7^, are needed to address the automatic lexico-semantic processing in the brain in more detail.

In sum, we demonstrate a neocortical signature of near-instant access to word representations in the brain: rapid neural dynamics that appears to arise in the temporo-frontal language network even when linguistic stimuli are presented outside the focus of attention. It shows specificity for familiar words starting as early as ~70 ms after the onset of unattended word stimuli in the temporal lobe, rapidly progresses to more anterior foci and concludes with an enhanced search in the brain’s mental lexicon when initial automatic parsing cannot be successful. The current results indicate early and automatic lexical processing of visually presented language in the brain that commences rapidly and may take place outside the focus of visual attention, similar to (and possible shared with) previously established mechanisms of spoken word access in the auditory modality. Thus, our brain is capable of near-instantaneous supra-modal access to language representations, a crucial capability for the efficient and reliable use of language as our primary communication tool.

## Methods

### Participants

Eighteen right-handed (according to the Edinburgh inventory^46^) native British English speakers (6 male, mean age 24 years; range 18–35 years) with normal vision and hearing and no record of neurological diseases took part in the experiments. Ethical approval was issued by Cambridge Psychology Research Ethics Committee (University of Cambridge). Informed written consent was obtained from all volunteers who were paid for their participation; the experiments were carried out in agreement with the Helsinki Declaration and in accordance with approved Good Research Practice guidelines (Medical Research Council, UK).

### Stimuli and Task

#### Language stimuli

108 distinct meaningful word stimuli were selected from the MRC Psycholinguistic Database of English language. All of these were short monosyllables that possessed a three-phoneme consonant-vowel-consonant (CVC) structure (e.g. lake, rope), consisted of 3–5 letters (mean 4.0) and had a familiarity rating of more than 300 to ensure that they are well known to native English speakers. They were accompanied by a set of 108 visually, orthographically and phonetically highly similar CVC pseudowords (e.g. *lape, *roke) that were matched with the words on the psycholinguistic properties of length, bigram and diphone frequencies. Note that all pseudowords were phonotactically legal and phonologically similar to words, which could be important to control in case of their covert articulation, even though the latter was highly unlikely given the task and procedures employed (see below).

#### Non-linguistic primary task stimuli

As a primary task, which the participants were instructed to concentrate on, they were presented with two circles of different colours (Fig. 1): all possible combinations of red, green, blue and yellow were used. These combinations were vertically arranged above and below the centrally-located crosshair and changed in synchrony with the orthographic stimuli that briefly appeared on visual periphery (see below). However, unlike the latter, the circles were kept on the screen for the entire duration of the SOA (to avoid strong visual onset and offset responses) such that they were seen as present continuously, only with their colours changing.

#### Task

The subjects were instructed to fixate their gaze on a fixation cross in the centre of projection screen, and to focus their attention on a dual visual task of detecting the features of the two circles, while ignoring everything else. This dual task required simultaneous tracking of the colours of both the upper and the lower circles and reacting only to a particular combination of the colours at specific locations. For example, for the task to respond to the combination of “upper red, lower blue” as a target, responses to any other combination – including “upper blue, lower red” – were considered incorrect. Responses to target combinations were given by pressing a response pad button with the left index finger. Probability of target combination appearance was 8%; within each recording session, stimulus sequences were randomised individually.

### Procedure

Prior to the recording, two bipolar vertical and horizontal electrooculogram (EOG) electrodes and four head position identification (HPI) coils were attached to the scalp. The HPI coil locations were digitised along with three fiducial points (nasion, left and right preauricular points) and 80 additional scalp locations (using Fastrak 3D digitiser, Polhemus, Colchester, VA) prior to MEG recording. The participants were then placed in a magnetically and acoustically shielded room (IMEDCO GMBH, Switzerland) and were instructed to stay calm and avoid movements and blinks as much as possible during the MEG recording.

They were instructed to fixate their gaze on a fixation cross in the centre of a projection screen, and to focus their attention on the dual visual task of simultaneously detecting colours of the two circles, while ignoring everything else. While the subjects concentrated on this primary task, unattended orthographic stimuli were presented at the left and right flanks as described below. The subjects were not informed of the orthographic stimuli, and the task did not encourage attention on them. On the contrary, the very brief presentation of these stimuli (100 ms) that appeared parafoveally at the same time as the colour combinations were changing in the attended primary task ensured maximum distraction from the textual stimulation.

The 216 orthographic stimuli (50% words, 50% pseudowords) were pseudorandomly presented for 100 ms, with stimulus onset asynchrony jittered between 1200–1800 ms (mean 1500 ms), in black font-face on grey background (Fig. 1, top). Using E-Prime software (Psychology Software Tools, Inc., Pittsburgh, PA, USA), two copies of each stimulus were simultaneously displayed at symmetric locations at 1.5° angle to the left and to the right from the centre of the screen. Such a symmetric bilateral presentation was used in order to ensure that while the complete information was presented to both visual hemifields, the participant’s gaze was not prompted to saccade from the primary task to the orthographic stimuli (the risk of which could be higher with a single asymmetric presentation). A short training sequence, using similar (but not identical) stimuli was run in the beginning of each recording session.

During the stimulation, neuromagnetic activity of the subjects’ brain was recorded (passband 0.03–330 Hz, sampling rate 1 kHz) continuously using 306-channel MEG setup (Elekta Neuromag, Helsinki, Finland). To control for eye movements, vertical and horizontal bipolar EOG were recorded along with the MEG data. To track the head position in the MEG helmet dewar, the position of four HPI coils was continuously monitored throughout the experiment using the continuous HPI (cHPI) recording protocol (Elekta Neuromag).

### Data processing

To minimise the contribution of magnetic sources from outside the head and to reduce any artifacts, the data from the 306 sensors were post-processed offline using a temporal extension of the Signal Space Separation (tSSS) method^47^ (as implemented in MaxFilter software, Elekta Neuromag; tSSS buffer duration of 4 s, correlation threshold of 0.98, inside and outside orders of expansion of 8 and 3, respectively, were used). Static bad channels were detected and excluded from subsequent processing steps, compensation was made for within-block head movements (as measured by HPI coils, with HPI step set to 200 ms) and externally generated artifacts were removed. For compatibility between different recordings, the data were converted to standard head position (x = 0 mm; y = 0 mm; z = 45 mm). Following this, epochs from 50 ms before to 800 ms after the onset of each stimulus were used for calculating event-related fields (ERFs) for the different stimulus types (word, pseudoword) using MNE Suite 2.7.0 software (Martinos Center for Biomedical Imaging, Charlestown, MA, USA). These were bandpass-filtered between 0.1 and 30 Hz and baseline-corrected using 50-ms prestimulus baseline. These settings follow a large body of previous MEG/EEG literature focussed on delineating early transient response dynamics. Epochs were rejected when the magnetic field variation at any gradiometer or magnetometer exceeded 3000 f T/cm or 6500 f T respectively, or when voltage variation at either bipolar EOG electrodes was greater than 150 μV. Remaining artefact-free epochs were averaged to produce individual event-related field responses separately for each condition and each subject.

High-resolution structural T1-weighted MRIs were acquired for each participant using a 3-Tesla Tim Trio MR scanner (Siemens, Erlangen, Germany; GRAPPA 3D MPRAGE sequence; TR = 2250 ms; TE = 2.99 ms; flip-angle = 9 degrees; acceleration factor = 2; 1 × 1 × 1 mm isotropic voxels). Cortical matter was segmented on the individual structural MRIs, and the estimated border between grey and white matter was tessellated. Individual single-layer boundary-element models (BEMs) were created for each participant using watershed segmentation algorithms (FreeSurfer 4.3 software, Martinos Center for Biomedical Imaging) to reconstruct the brain’s cortical grey matter surface as a high-resolution triangularised mesh with 10424 vertices in each hemisphere. The surface was further ‘inflated’ to unfold cortical sulci to provide their optimal view. Cortical sources of the observed neuromagnetic activity were computed using the MNE Suite 2.7.0 software based on signals from all 306 MEG sensors and the L2 Minimum-Norm Estimates (MNE) algorithm which models the recorded magnetic field distribution with the smallest amount of overall source activity^48^,^49^.

For signal-space analysis, a cluster of 16 gradiometers (8 sets with 2 planar gradiometers in each) broadly covering left perisylvian language cortices was used, in line with previous neurolinguistic studies using similar MEG sensor configurations. This was subdivided into frontal and temporal regions-of-interest (ROIs). In each sensor ROI, aggregated neuromagnetic activation was quantified by computing the square root of the mean of squared amplitudes of all gradiometer pairs (RMS, root mean square). This technique, most commonly used for analysing planar gradiometer data, not only provides a timecourse of the mass neuronal activity registered in MEG, but also offers an advantage over other types of non-invasive recordings (such as EEG, magnetometer or axial gradiometer data) in displaying the strongest amplitude directly above the cortical source, thus providing additional reassurance with respect to the spatial origin of any effects. Here, the most prominent event-related responses arose around 80, 150 and 230 ms. These were therefore taken to further analysis using window-average amplitudes extracted from 20-ms wide windows set at 70–90 ms, 140–160 ms and 220–240 ms.

Given the inevitably crude definition of the sensor ROIs and especially the variability in their location with respect to individual heads across the participant group, the interpretation of sensor-level ROI activation as “temporal” or “frontal” is tentative and should be treated with caution (although this critique does not undermine any *relative* differences between the more posterior and more anterior sensor clusters). Sensor level effects were therefore followed up by source analysis. For the comparison of source strength, cortical regions were selected based on the Desikan-Killiany parcellation of the cortical surface as implemented in the FreeSurfer package^50^. We a priori focused our analysis on activity in two classic regions which are well known to contribute to language processing: temporal and inferior frontal cortices (Fig. 1C). Other areas remained outside the scope of this study. Regions included in the temporal ROI were superior, middle and inferior temporal gyri subdivided into anterior and posterior parts, while the frontal ROI included pars opercularis, pars triangularis and pars orbitalis (traditionally considered to compose the Broca area).

For the statistical analysis, identical approach was used for assessing effects in both sensor signal and source spaces. Mean amplitudes of the ERFs (in signal space) or source currents (in source space) were calculated over the time windows of interest defined in the sensor-level analysis above, and entered into an analysis of variance analysis with factors Lexicality (word vs. pseudoword), ROI (temporal vs. frontal) and Hemisphere (left vs. right). Significant interactions were followed up by planned comparisons; where significant results were found at the main ROI level, a further analysis was done employing the sub-ROI factor taking into account finer-grain neuroanatomical partitioning of the frontal and temporal ROIs, as described above.

## Additional Information

**How to cite this article**: Shtyrov, Y. and MacGregor, L. J. Near-instant automatic access to visually presented words in the human neocortex: neuromagnetic evidence. *Sci. Rep.* **6**, 26558; doi: 10.1038/srep26558 (2016).
